# Supplementary material for: Shu complex SWS1-SWSAP1 promotes early steps in mouse meiotic recombination
Source: Nat Commun. 2018 Oct 10;9:3961. doi: 10.1038/s41467-018-06384-x (PMC6180034; doi:10.1038/s41467-018-06384-x)
Supplement: Supplementary file 1 — Supplementary Information [file 41467_2018_6384_MOESM1_ESM.pdf]

Shu complex SWS1-SWSAP1 promotes early steps in mouse meiotic recombination

Abreu et al.

Supplementary Information

Supplementary Figure 1

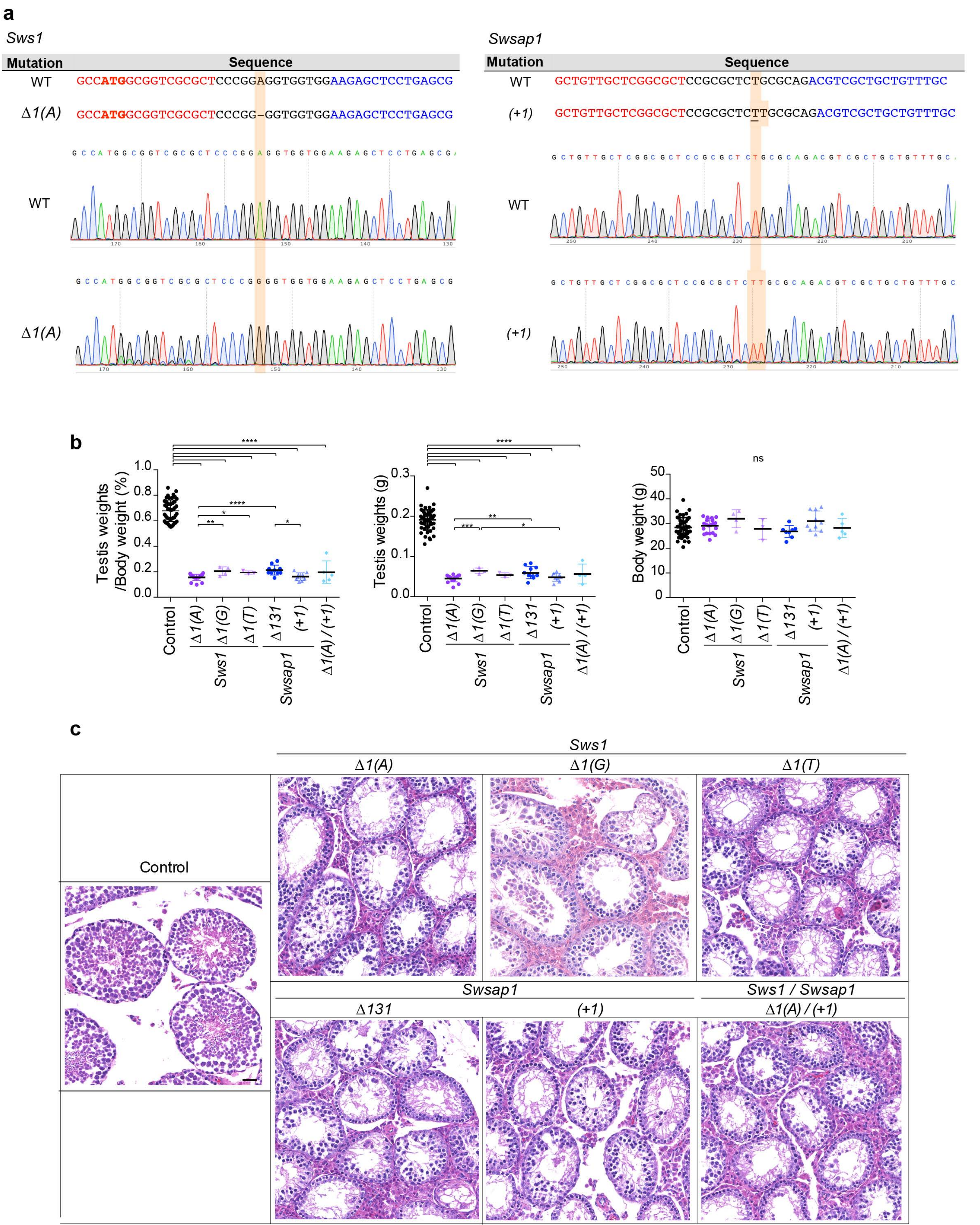

Supplementary Figure 1: Meiotic defects in *Sws1* and *Swsap1* mutant testes.

(a) RT-PCR was performed to confirm mutant alleles, as specific antibodies for SWS1 and SWSAP1 could not be confirmed by western blot analysis. Sanger sequencing of cDNA from testis of *Sws1Δ1(A)* and *Swsap1(+1)* mice demonstrates the expected 1-bp deletion and 1-bp addition, respectively, in transcripts from these alleles. Positions of these mutations are highlighted in orange. n=2.

(b) All Shu complex single and double mutants have similarly reduced testis to body weight ratios and testis weights but not body weights. The testis to body weight ratios are from the combined graph in Fig. 1**c** but here are separated according to allele. Error bars: mean±s.d. Mice: Control, n=45; $Sws1^{-/-}$ [Δ*1(A*), n=20; Δ*1(G)*, n=4; Δ*1(T)*, n=3]; $Swsap1^{-/-}$ [Δ*131*, n=10; *(+1)*, n=8]; $Sws1^{-/-}$ $Swsap1^{-/-}$ (Δ*1(A)* / *(+1)*), n=5. ns, not significant compared to control; *, $P \le 0.05$; **, $P \le 0.01$; ***, $P \le 0.001$; ****, $P \le 0.0001$; Student's *t*-test, two-tailed.

(c) Spermatocytes from single and double mutants show meiotic arrest. Testis sections were stained with H&E. Scale bars, 100 μm. n≥2.

Supplementary Figure 2

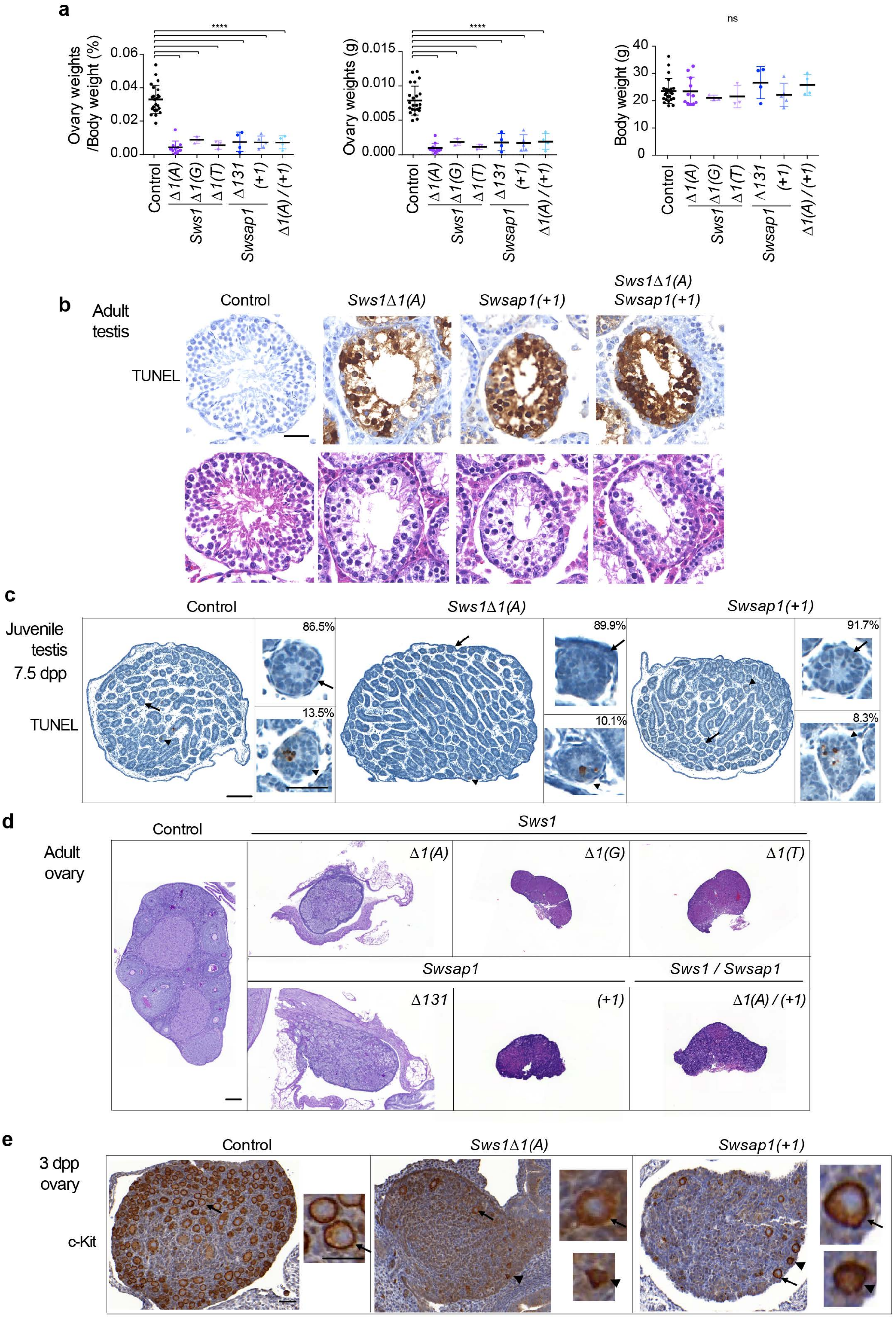

Supplementary Figure 2: Shu mutants have abnormal ovaries and adult testes.

(a) Shu complex single and double mutants have reduced ovary to body weight ratios and ovary weights but not body weights. The ovary to body weight ratios are from the combined graph in Fig. 1**d** but here are separated according to allele. Error bars: mean±s.d. Mice: Control, n=23; *Sws1*$^{-/-}$ [Δ*1(A)*, n=11; Δ*1(G)*, n=3; Δ*1(T)*, n=3]; *Swsap1*$^{-/-}$ [Δ*131*, n=4; *(+1)*, n=5]; *Sws1*$^{-/-}$ *Swsap1*$^{-/-}$ (Δ*1(A) / (+1)*), n=4. ns, not significant compared to control; ****, $P \leq 0.0001$, significant compared to control; Student's *t*-test, two-tailed.

(b) Widespread apoptosis is observed in seminiferous tubules from single- and double-mutant testes in adults. Sections were stained with hematoxylin and TUNEL was used to visualize apoptotic spermatocytes. Scale bar, 50 µm. n=2.

(c) Apoptosis in 7.5-dpp *Sws1*$^{-/-}$ and *Swsap1*$^{-/-}$ juvenile testes is rarely observed indicating that premeiotic stages are not grossly affected. Arrowheads point to rare apoptotic cells in both control and Shu mutants. Insets indicate the % TUNEL-negative (arrows) and positive (arrowheads) tubules. Sections were stained and analyzed as in **b**. Scale bar, 50 µm and 20 µm insets. n=2.

(d) Adult ovaries from single and double mutants show an absence of follicles at all stages of oocyte development. Sections were stained with H&E. Scale bar, 500 µm. n≥2.

(e) Ovaries from 3-dpp *Sws1*$^{-/-}$ and *Swsap1*$^{-/-}$ mice show significantly reduced primordial and primary follicles, some of which appeared to be apoptotic based on the condensed chromatin. Insets show non-apoptotic (arrows) and possibly apoptotic (arrowheads) follicles. Sections were stained with c-Kit. Scale bar, 100 µm and 20 µm insets. n=2.

Supplementary Figure 3

a

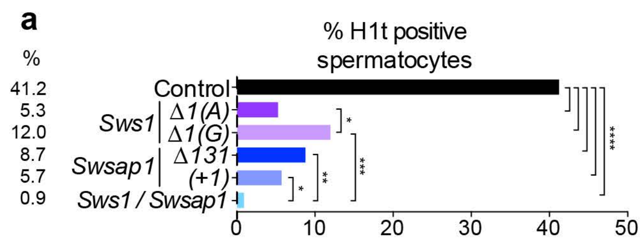

b

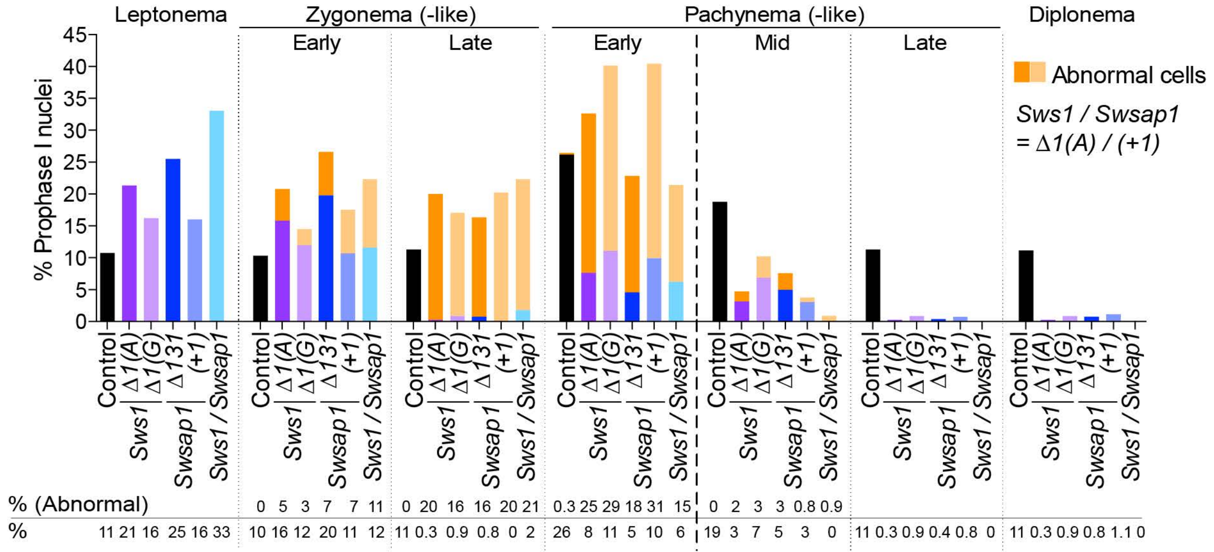

c

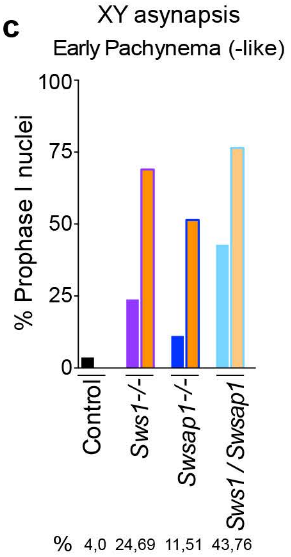

d

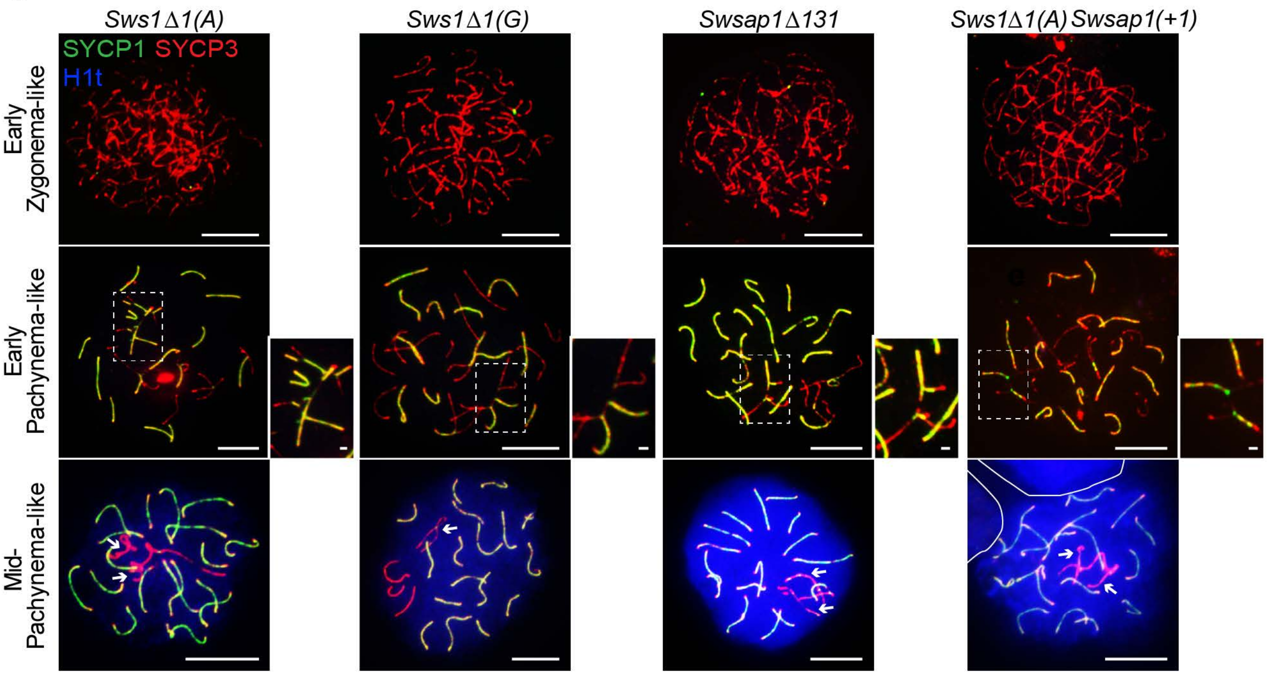

e

Early-pachytene(-like) cells with non-homologous synapsis

Mice	%
Control	3.6
Sws1Δ1(A)	44.4
Sws1Δ1(G)	51.1
Swsap1Δ131	38.3
Swsap1(+1)	35.9
Sws1Δ1(A) Swsap1(+1)	37.5

f

Pachytene(-like) cells with chromosomal end-to-end fusions

Mice	%
Control	7.8
Sws1Δ1(A)	21.0
Sws1Δ1(G)	26.7
Swsap1Δ131	18.5
Swsap1(+1)	9.8
Sws1Δ1(A) Swsap1(+1)	12.0

Supplementary Figure 3: Spermatocytes from Shu complex mutant mice have defects in meiotic progression.

(a) Histone H1t staining indicates that few spermatocytes in *Sws1* and *Swsap1* single and double mutants progress to mid-pachynema and beyond. Total number of mid-pachytene, late-pachytene, and diplotene spermatocytes divided by the total number of spermatocytes analyzed from adult testes. Mice: Control, *Sws1*$^{-/-}$Δ*1(A)*, *Swsap1*$^{-/-}$Δ*131*, from Fig. 2**a**; *Sws1*$^{-/-}$Δ*1(G), Sws1*$^{-/-}$ *Swsap1*$^{-/-}$ (Δ*1(A) / (+1)),* n=1; *Swsap1*$^{-/-}$*(+1)*, n=2. *, $P \leq 0.05$; **, $P \leq 0.01$; ***, $P \leq 0.001$; ****, $P \leq 0.0001$; Fisher's exact test, two-tailed.

(b-f) *Sws1* and *Swsap1* mutants show altered meiotic progression and abnormal synapsis of both autosomes and sex chromosomes. Percentage of spermatocytes in each of the indicated meiosis prophase I stages is shown in **b,** with the percentage of early pachytene (-like) spermatocytes with XY asynapsis in **c**. Mice are the same as those in **a** (except for *Swsap1(+1)*, n=1 in **c**,**e**,**f**), and are combined for each mutant in **c**. Spermatocytes were staged as in Fig. 2**b**. The subset of cells with autosomal synapsis defects are summarized with the orange bars. Representative chromosome spreads of abnormal spermatocytes at early zygonema, and early and mid-pachynema are shown in **d**; percentages of early-pachytene (-like) cells with non-homologous synapsis are in **e**; and percentages of pachytene (-like) cells with chromosomal end-to-end fusions are in **f**. Boxes and respective insets at early pachynema highlight non-homologous synapsis that is often in combination with homologous synapsis and partial asynapsis, resulting in chromosome tangles; arrows at mid-pachynema indicate unsynapsed chromosomes. Spermatocytes with complete homolog synapsis but chromosome end-to-end fusions are considered normal for this analysis. Scale bars in zoomed-out images, 10 μm. Scale bars in insets, 1 μm.

Supplementary Figure 4

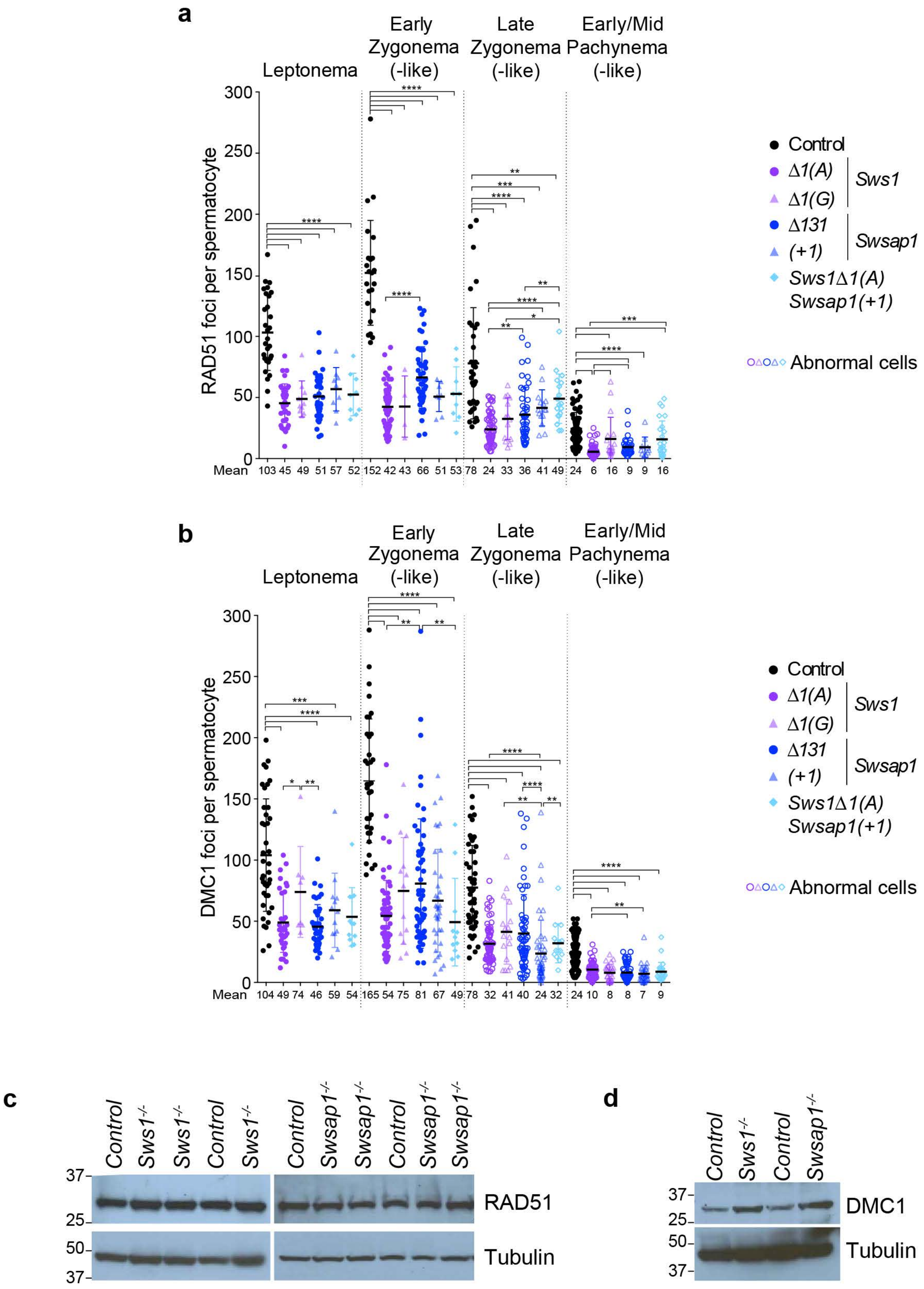

Supplementary Figure 4: SWS1 and SWSAP1 are required for RAD51 and DMC1 focus assembly during meiosis.

(a,b) RAD51 and DMC1 focus counts are reduced in *Sws1* and *Swsap1* mutant spermatocytes. Each symbol is the total number of foci from a single nucleus. Solid symbol, normal cells. Open symbol, abnormal cells. Error bars, mean±s.d. *, $P \leq 0.05$; **, $P \leq 0.01$; ***, $P \leq 0.001$; ****, $P \leq 0.0001$; Mann-Whitney test, one-tailed. Mice: Control, $Sws1^{-/-}$Δ*1(A)*, $Swsap1^{-/-}$Δ*131*, from Fig. 2**e**,**g**; $Sws1^{-/-}$Δ*1(G)*, $Swsap1^{-/-}$*(+1)*, $Sws1^{-/-}$ $Swsap1^{-/-}$ (Δ*1(A) / (+1)*), n=1.

(c,d) RAD51 and DMC1 protein levels are similar in *Sws1* and *Swsap1* mice to control mice. For RAD51: Control, n=4; $Sws1^{-/-}$ Δ*1(A)*, n=3; $Swsap1^{-/-}$ Δ*131*, n=4. For DMC1: Control, n=2; $Sws1^{-/-}$ Δ*1(A)*; $Swsap1^{-/-}$ Δ*131*, n=1.

Supplementary Figure 5

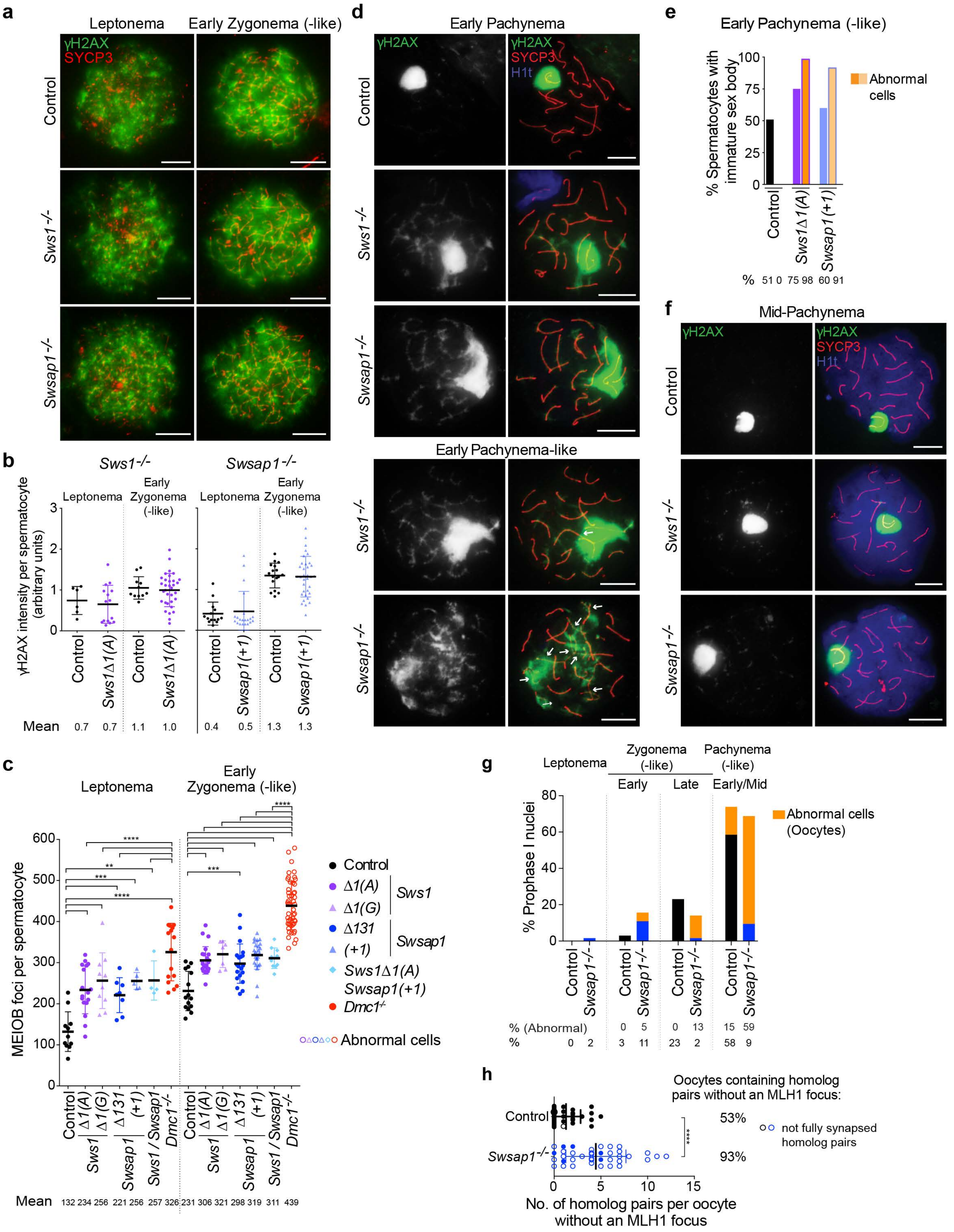

Supplementary Figure 5: DSB formation appears to be normal in *Sws1* and *Swsap1* mutant spermatocytes, while DSB repair is compromised.

(a,b) DSB formation, as measure by γH2AX signal intensity levels, is unaffected in *Sws1*$^{-/-}$ and *Swsap1*$^{-/-}$ spermatocytes. Representative chromosome spreads at the indicated stages from adult mice in **a** with γH2AX integrated density in **b**. Scale bars, 10 µm. Each symbol in **b** is from a single nucleus to which the average background signal from four regions around each nucleus was subtracted. Error bars, mean±s.d. Mice: Control, *Sws1*$^{-/-}$Δ*1(A)*, *Swsap1*$^{-/-}$*(+1)*, n=1.

(c) MEIOB focus counts are increased in *Sws1*$^{-/-}$ and *Swsap1*$^{-/-}$ spermatocytes, but not as high as in *Dmc1*$^{-/-}$ spermatocytes. Each symbol is the total focus number from a single nucleus. Solid symbol, normal cells. Open symbol, abnormal cells. Error bars, mean±s.d. Mice: Control, *Sws1*$^{-/-}$ Δ*1(A)*, *Swsap1*$^{-/-}$Δ*131*, *Dmc1*$^{-/-}$, from Fig. 3**b**; *Sws1*$^{-/-}$Δ*1(G)*, *Swsap1*$^{-/-}$*(+1)*, *Sws1*$^{-/-}$ *Swsap1*$^{-/-}$ (Δ*1(A) / (+1))*, n=1. **, *P*≤0.01; ***, *P*≤0.001; ****, *P*≤0.0001; Mann-Whitney test, one-tailed.

(d-f) At early pachynema, *Sws1*$^{-/-}$ and *Swsap1*$^{-/-}$ spermatocytes show unrepaired DSBs associated with defects in sex body formation/maturation, whereas the small fraction of mid-pachytene mutant spermatocytes are apparently more repair proficient. Representative chromosome spreads at the indicated stages in **d**,**f** with percentage of nuclei displaying immature sex body in **e**. Arrows indicate unsynapsed chromosomes. Scale bars, 10 µm. Control, n=3; *Sws1*$^{-/-}$Δ*1(A)*, *Swsap1*$^{-/-}$*(+1)*, n=2.

(g,h) Most oocytes from control and *Swsap1* mice at embryonic day 18.5 have entered pachynema with the majority of mutant oocytes showing synapsis defects and one or more bivalents without an MLH1 focus. Percentage of oocytes at each of the indicated stages in **g** with the number of homolog pairs that are not synapsed and/or are lacking an MLH1 focus in **h**. The total percentage of oocytes containing homolog pairs without an MLH1 focus is also indicated. Error bars in **h**, mean±s.d. Solid circles, oocytes with fully synapsed homologs. Open circles, abnormal oocytes containing homologs partially synapsed and/or one or two homolog pairs fully unsynapsed. Mice from Fig. 3**i**. ****, *P*≤0.0001; Mann-Whitney test, one-tailed.

Supplementary Figure 6

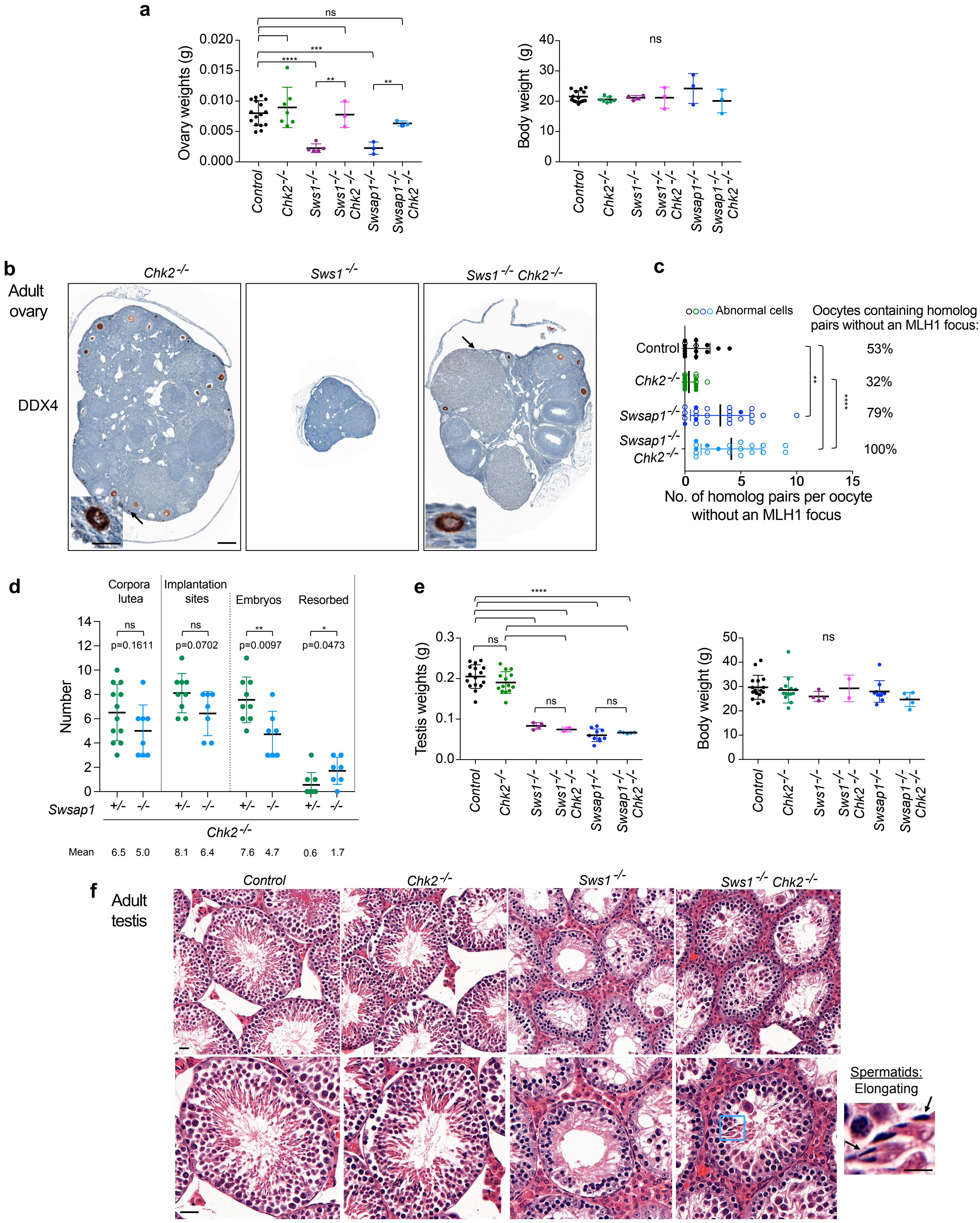

Supplementary Figure 6: Effect of *Chk2* mutation on ovary and/or testis weights in *Sws1* and *Swsap1* mutant mice.

(a) Ovary weights of *Sws1*$^{-/-}$ *Chk2*$^{-/-}$ and *Swsap1*$^{-/-}$ *Chk2*$^{-/-}$ mice are significantly higher than those of *Sws1*$^{-/-}$ and *Swsap1*$^{-/-}$ mice with no significant difference in the body weight. Mice: Control, n=16; *Chk2*$^{-/-}$, n=7; *Sws1*$^{-/-}$Δ*1(A),* n=5; *Sws1*$^{-/-}$Δ*1(A) Chk2*$^{-/-}$, n=3; *Swsap1*$^{-/-}$, *Swsap1*$^{-/-}$ *Chk2*$^{-/-}$, n=3. **, $P \leq 0.01$; ***, $P \leq 0.001$; ****, $P \leq 0.0001$; Student's *t*-test; two-tailed.

(b) Adult *Sws1*$^{-/-}$ *Chk2*$^{-/-}$ ovaries have follicles at various stages of oocyte development, unlike *Sws1*$^{-/-}$ ovaries. Primordial follicles in *Chk2*$^{-/-}$ and *Sws1*$^{-/-}$ *Chk2*$^{-/-}$ are highlighted in insets and by arrows. Sections were stained with hematoxylin and an antibody for DDX4/Vasa. Scale bar, 500 µm and 50 µm insets. Mice: *Chk2*$^{-/-}$, *Sws1*$^{-/-}$Δ*1(A)* and *Sws1*$^{-/-}$Δ*1(A) Chk2*$^{-/-}$, n=3.

(c) All *Swsap1*$^{-/-}$ and *Swsap1*$^{-/-}$ *Chk2*$^{-/-}$ oocytes from mice at embryonic day 18.5 have one or more homologs fully unsynapsed and/or lacking one MLH1 focus. The total percentage of oocytes containing homologs without an MLH1 focus is also indicated. Solid circles, oocytes with homologs fully synapsed. Open circles, abnormal oocytes with homologs partially synapsed and/or one or more homolog pairs fully unsynapsed. Mice from Fig. 4e. Data for control and *Swsap1*$^{-/-}$ are from Supplementary Fig. 5h. **, $P \leq 0.01$; ****, $P \leq 0.0001$; Mann-Whitney test, one-tailed.

(d) Pregnant *Swsap1*$^{-/-}$ *Chk2*$^{-/-}$ females at 12.5 dpc have a significant reduction in the number of embryos and an increase in the number resorbed embryos relative to *Swsap1*$^{+/-}$ *Chk2*$^{-/-}$ females. The number of corpora lutea (an estimate of ovulated oocyte numbers) were counted in ovary sections and implantation sites (distinguishing between those with normal versus resorbed embryos) were counted in uterine horns. Corpora lutea: *Swsap1*$^{+/-}$ *Chk2*$^{-/-}$, n=12; *Swsap1*$^{-/-}$ *Chk2*$^{-/-}$, n=8. Implantation sites, embryos and resorbed: *Swsap1*$^{+/-}$ *Chk2*$^{-/-}$, n=9; *Swsap1*$^{-/-}$ *Chk2*$^{-/-}$, n=7. *, $P \leq 0.05$; **, $P \leq 0.01$; Student's *t*-test, two-tailed.

(e) Testis weights and body weights of *Sws1*$^{-/-}$ *Chk2*$^{-/-}$ and *Sws1*$^{-/-}$, *Swsap1*$^{-/-}$ *Chk2*$^{-/-}$ and *Swsap1*$^{-/-}$ mice are similar. Mice: Control, n=15; *Chk2*$^{-/-}$, n=14; *Sws1*$^{-/-}$Δ*1(A),* n=4; *Sws1*$^{-/-}$Δ*1(A) Chk2*$^{-/-}$, n=2; *Swsap1*$^{-/-}$, n=10; *Swsap1*$^{-/-}$ *Chk2*$^{-/-}$, n=5. ****, $P \leq 0.0001$; Student's *t*-test, two-tailed.

(f) Seminiferous tubules in adult *Sws1*$^{-/-}$ *Chk2*$^{-/-}$ mice occasionally contain elongating spermatids (blue rectangles and insets). Sections were stained with H&E. Scale bars, 100 µm (top panel), 50 µm (bottom panel), and 20 µm (inset).

Error bars in **a**,**c**,**d**,**e**: mean±s.d.; ns, not significant compared to control.

Supplementary Figure 7

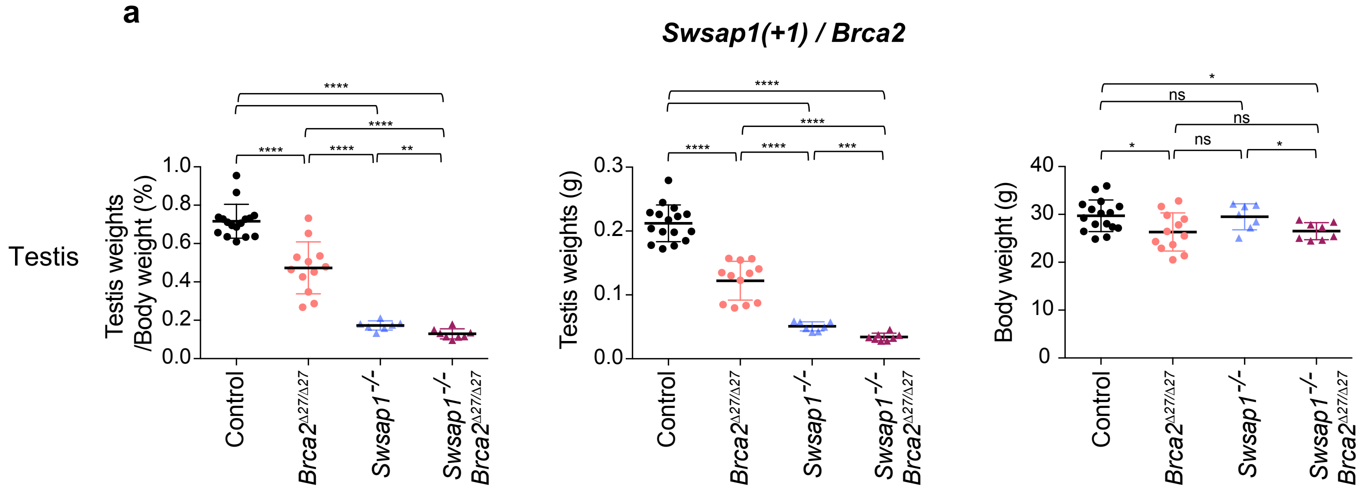

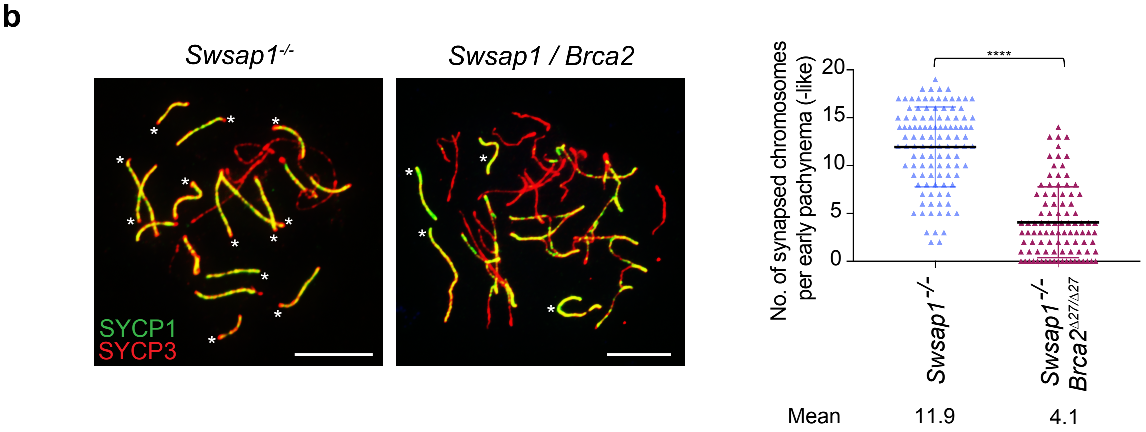

Supplementary Figure 7: Effect of *Brca2*$^{\Delta 27}$ mutation on testis weights in *Swsap1* mice.

(a) Testis to body weight ratios and testis weights of *Swsap1*$^{-/-}$*(+1) Brca2*$^{\Delta 27/\Delta 27}$ are 1.3- and 1.7-fold reduced respectively compared to *Swsap1*$^{-/-}$*(+1)* mice but no major difference in the body weights is seen in these mice. Mice: Control, n=16; *Brca2*$^{\Delta 27/\Delta 27}$, n=12; *Swsap1*$^{-/-}$*(+1),* n=7; *Swsap1*$^{-/-}$*(+1) Brca2*$^{\Delta 27/\Delta 27}$, n=8. ns, not significant; *, $P \leq 0.05$; **, $P \leq 0.01$; ***, $P \leq 0.001$; ****, $P \leq 0.0001$; Student's *t*-test, two-tailed.

(b) The number of synapsed chromosomes in *Swsap1*$^{-/-}$*(+1) Brca2*$^{\Delta 27/\Delta 27}$ mutants is 3-fold reduced compared to *Swsap1*$^{-/-}$*(+1)* spermatocytes. Representative chromosome spreads from early pachytene-like cells are shown. Scale bars, 10 µm. n=3. ****, $P \leq 0.0001$; Mann-Whitney, one-tailed.

Error bars: mean±s.d.

Supplementary Table 1a

Sws1 mutational analysis

Mouse	Mutation	μ hom	Sequence
WT	WT		CGGGCGGCGTGCCATGGCGGTCGCGCTCCCGGAGGTGGTGGAAGAGCTCCTGAGCG
13	Δ1		CGGGCGGCGTGCCATGGCGGTCGCGCTCCCGGAGGTGGTGGAAGAGCTCCTGAGCG
13	Δ1	1bp	CGGGCGGCGTGCCATGGCGGTCGCGCTCCCGGAGGTGGTGGAAGAGCTCCTGAGCG
42	Δ1		CGGGCGGCGTGCCATGGCGGTCGCGCTCCCGGAGGTGGTGGAAGAGCTCCTGAGCG
42	Δ3		CGGGCGGCGTGCCATGGCGGTCGCGCTCCCGGAGGTGGTGGAAGAGCTCCTGAGCG
43	Δ1	1bp	CGGGCGGCGTGCCATGGCGGTCGCGCTCCCGGAGGTGGTGGAAGAGCTCCTGAGCG
43	Δ18 & +7		CGGGCGGCGTGCCATGGCGGTCGCGCTCCCGGAGCCCAAAAGTGGTGGAAGAGCTCCTGAGCG
30	Δ2	1bp	CGGGCGGCGTGCCATGGCGGTCGCGCTCCCGGAGGTGGTGGAAGAGCTCCTGAGCG
30	Δ21	2bp	CGGGCGGCGTGCCATGGCGGTCGCGCTCCCGGAGGTGGTGGAAGAGCTCCTGAGCG
5	Δ3	3bp	CGGGCGGCGTGCCATGGCGGTCGCGCTCCCGGAGGTGGTGGAAGAGCTCCTGAGCG
5	Δ15	3bp	CGGGCGGCGTGCCATGGCGGTCGCGCTCCCGGAGGTGGTGGAAGAGCTCCTGAGCG
5	Δ21	3bp	CGGGCGGCGTGCCATGGCGGTCGCGCTCCCGGAGGTGGTGGAAGAGCTCCTGAGCG
29	Δ3	3bp	CGGGCGGCGTGCCATGGCGGTCGCGCTCCCGGAGGTGGTGGAAGAGCTCCTGAGCG
29	Δ6	2bp	CGGGCGGCGTGCCATGGCGGTCGCGCTCCCGGAGGTGGTGGAAGAGCTCCTGAGCG
29	Δ20		CGGGCGGCGTGCCATGGCGGTCGCGCTCCCGGAGGTGGTGGAAGAGCTCCTGAGCG
40	Δ9	2bp	CGGGCGGCGTGCCATGGCGGTCGCGCTCCCGGAGGTGGTGGAAGAGCTCCTGAGCG
27	Δ6	2bp	CGGGCGGCGTGCCATGGCGGTCGCGCTCCCGGAGGTGGTGGAAGAGCTCCTGAGCG
47	Δ6	2bp	CGGGCGGCGTGCCATGGCGGTCGCGCTCCCGGAGGTGGTGGAAGAGCTCCTGAGCG
47	Δ21	3bp	CGGGCGGCGTGCCATGGCGGTCGCGCTCCCGGAGGTGGTGGAAGAGCTCCTGAGCG
23	Δ18	3bp	CGGGCGGCGTGCCATGGCGGTCGCGCTCCCGGAGGTGGTGGAAGAGCTCCTGAGCG
41	Δ18	3bp	CGGGCGGCGTGCCATGGCGGTCGCGCTCCCGGAGGTGGTGGAAGAGCTCCTGAGCG
38	Δ18	3bp	CGGGCGGCGTGCCATGGCGGTCGCGCTCCCGGAGGTGGTGGAAGAGCTCCTGAGCG
38	Δ24	3bp	CGGGCGGCGTGCCATGGCGGTCGCGCTCCCGGAGGTGGTGGAAGAGCTCCTGAGCG
20	Δ21	3bp	CGGGCGGCGTGCCATGGCGGTCGCGCTCCCGGAGGTGGTGGAAGAGCTCCTGAGCG
46	Δ21	3bp	CGGGCGGCGTGCCATGGCGGTCGCGCTCCCGGAGGTGGTGGAAGAGCTCCTGAGCG
31	Δ21	3bp	CGGGCGGCGTGCCATGGCGGTCGCGCTCCCGGAGGTGGTGGAAGAGCTCCTGAGCG
22	Δ21	3bp	CGGGCGGCGTGCCATGGCGGTCGCGCTCCCGGAGGTGGTGGAAGAGCTCCTGAGCG
22	Δ45	3bp	CGGGCGGCGTGCCATGGCGGTCGCGCTCCCGGAGGTGGTGGAAGAGCTCCTGAGCGAG
36	Δ21	2bp	CGGGCGGCGTGCCATGGCGGTCGCGCTCCCGGAGGTGGTGGAAGAGCTCCTGAGCG
36	Δ27		CGGGCGGCGTGCCATGGCGGTCGCGCTCCCGGAGGTGGTGGAAGAGCTCCTGAGCG
15	Δ24	3bp	CGGGCGGCGTGCCATGGCGGTCGCGCTCCCGGAGGTGGTGGAAGAGCTCCTGAGCG
39	Δ24	3bp	AGAAGACGGGCGGCGTGCCATGGCGGTCGCGCTCCCGGAGGTGGTGGAAGAGCTCCTGAGCG
39	Δ45	1bp	AGAAGACGGGCGGCGTGCCATGGCGGTCGCGCTCCCGGAGGTGGTGGAAGAGCTCCTGAGCG
19	Δ31	1bp	CGGGCGGCGTGCCATGGCGGTCGCGCTCCCGGAGGTGGTGGAAGAGCTCCTGAGCG
19	Δ30		CaGGCGGCGTGCCATGGCGGTCGCGCTCCCGGAGGTGGTGGAAGAGCTCCTGAGCG
24	Δ34	2bp	CGGGCGGCGTGCCATGGCGGTCGCGCTCCCGGAGGTGGTGGAAGAGCTCCTGAGCG

Supplementary Table 1b

Swsap1 mutational analysis

Mouse	Mutation	μ hom	sequence
WT			GCTGTTGCTCGGCGCTCCGCGCTCTGCGCAGACGTCGCTGCTGTTTGC
18	+1	1bp	GCTGTTGCTCGGCGCTCCGCGCTCTTGCGCAGACGTCGCTGCTGTTTGC
23	+1	1bp	GCTGTTGCTCGGCGCTCCGCGCTCTTGCGCAGACGTCGCTGCTGTTTGC
29	Δ1	0	GCTGTTGCTCGGCGCTCCGCGCTCTGCGCAGACGTCGCTGCTGTTTGC
45	Δ2	0	GCTGTTGCTCGGCGCTCCGCGCTCTGCGCAGACGTCGCTGCTGTTTGC
13	Δ2	2bp	GCTGTTGCTCGGCGCTCCGCGCTCTGCGCAGACGTCGCTGCTGTTTGC
13	Δ73	0	GCTGTTGCTCGGCGCTCCGCGCTCTGCGCAGACGTCGCTGCTGTTTGC
5	Δ6	1bp	GCTGTTGCTCGGCGCTCCGCGCTCTGCGCAGACGTCGCTGCTGTTTGC
27	Δ18	3bp	GCTGTTGCTCGGCGCTCCGCGCTCTGCGCAGACGTCGCTGCTGTTTGC
12	Δ32	0	GCTGTTGCTCGGCGCTCCGCGCTCTGCGCAGACGTCGCTGCTGTTTGC
12	Δ87	0	GCTGTTGCTCGGCGCTCCGCGCTCTGCGCAGACGTCGCTGCTGTTTGC
32	Δ39	7bp	TGCTGTTGCTCGGCGCTCCGCGCTCTGCGCAGACGTCGCTGCTGTTTGC
32	Δ32	7bp	TGCTGTTGCTCGGCGCTCCGCGCTCTGCGCAGACGTCGCTGCTGTTTGC
33	Δ132 & +1	6bp	GCTGTTGCTCGGCGCTCCGCGCTCTGCGCAGACGTCGCTGCTGTTTGC

Supplementary Table 1 legend:
Sws1 and *Swsap1* founder mice obtained after TALEN expression in **a** and **b**, respectively. Left and right TALEN binding sites are shown in red and blue. Indels are highlighted in light green. μhom refers to the microhomology at the breakpoint junction (underlined). Each *Sws1* founder mouse contained a wild-type allele except for 15, 20, 23, 27, 43 and 46. Each *Swsap1* founder mouse contained a wild-type allele. Forty-seven potential founders were born for *Sws1*, of which 22 were further analyzed based on T7 assays; 20 different indels were identified in these founder mice, of which 6 were frame-shift mutations. Forty-eight potential founders were born for *Swsap1*, of which 10 were further analyzed; 12 different indels were identified, of which 8 were frame-shift mutations.

Supplementary Table 2

a

	**Mutant allele**	**No. of mice**	**+/+**	**+/-**	**-/-**	**P-values**
Sws1	*Sws1 Δ1(A)*	256	86 (34%)	118 (46%)	52 (20%)	0.0050
	Sws1 Δ1(G)	174	48 (28%)	84 (48%)	42 (24%)	0.7332
	Sws1 Δ1(T)	51	11 (22%)	29 (56%)	11 (22%)	0.6185
Swsap1	*Swsap1 Δ131*	215	53 (24%)	107 (50%)	55 (26%)	0.9793
	Swsap1 (+1)	262	77 (29%)	138 (53%)	47 (18%)	0.0222

b

	Control				$Sws1^{-/-}$		$Swsap1^{-/-}$		$Sws1^{-/-}$ $Swsap1^{-/-}$
Sws1 / Swsap1	*+/+; +/+*	*Δ1(A)/+; +/+*	*+/+; (+1)/+*	*Δ1(A)/+; (+1)/+*	*Δ1(A)/Δ1(A); +/+*	*Δ1(A)/Δ1(A); (+1)/+*	*+/+; (+1)/(+1)*	*Δ1/+; (+1)/(+1)*	*Δ1(A)/Δ1(A); (+1)/(+1)*
Observed	20 (7.1%)	37 (13.1%)	34 (12.0%)	78 (27.7%)	16 (5.7%)	36 (12.8%)	15 (5.3%)	33 (11.7%)	13 (4.6%)
Expected	6.25%	12.50%	12.50%	25.00%	6.25%	12.5%	6.25%	12.50%	6.25%

n=282

Supplementary Table 2 legend: $Sws1^{-/-}$, $Swsap1^{-/-}$ and double mutant mice are viable. Heterozygous mice for each genotype were bred to obtain homozygous knockouts. *P*-values were obtained using Chi squared analysis. Although *Sws1Δ1(A) and Swsap1(+1)* mutants appeared to be underrepresented in **a**, they were represented at the normal Mendelian ratio in **b**.

Supplementary Table 3

a

Sws1 ♂ x ♀	No. of litters	Avg. no.pups per litter	P-values
+/- x +/-	3	5.7	
+/- x +/-	3	7	
-/- x +/-	0	-	*P*≤0.0001
-/- x +/-	0	-	
+/- x -/-	0	-	*P*≤0.0001
+/- x -/-	0	-	

Swsap1 ♂ x ♀	No. of litters	Avg. no.pups per litter	P-values
+/- x +/-	3	5.7	
+/- x +/-	3	7	
-/- x +/-	0	-	*P*≤0.0001
-/- x +/-	0	-	
+/- x -/-	0	-	*P*≤0.0001

b

Swsap1 / Chk2 ♂ x ♀	No. of litters	Avg. no.pups per litter	P-value
+/-; -/- x +/-; -/-	4	6	
+/-; -/- x +/-; -/-	3	6.5	
+/-; -/- x -/-; -/-	3	3	*P*=0.0047
+/-; -/- x -/-; -/-	3	3	
+/-; -/- x -/-; -/-	3	4	
+/-; -/- x -/-; -/-	3	3	

Double-mutant dams are themselves offspring of double-mutant dams.

Swsap1 / Chk2 ♂ x ♀	No. of litters	Avg. no.pups per litter	P-value
+/-; -/- x +/-; -/-	4	6	
-/-; -/- x +/+; +/+	0	-	*P*≤0.0001
-/-; -/- x +/+; +/+	0	-	

c

Sws1 / Chk2 ♂ x ♀	No. of litters	no. of pups per litter
+/-; -/- x +/-; -/-	1	7
+/-; -/- x -/-; -/-	1	4

Supplementary Table 3:
Fertility assessment of $Sws1^{-/-}$ and $Swsap1^{-/-}$ in **a** and $Swsap1^{-/-}Chk2^{-/-}$, $Sws1^{-/-}Chk2^{-/-}$ in **b** and **c** respectively. The single- or double-mutant mice (shown in red) were bred with heterozygous or wild-type mice for 4-5 months. Control heterozygous breedings were set up for the same period of time. *P*-values were obtained using Mann-Whitney test, two-tailed.
