# Supplementary material for: Shu complex SWS1-SWSAP1 promotes early steps in mouse meiotic recombination
Source: Nat Commun. 2018 Oct 10;9:3961. doi: 10.1038/s41467-018-06384-x (PMC6180034; doi:10.1038/s41467-018-06384-x)
Supplement: Supplementary file 3 — Description of Additional Supplementary Files [file 41467_2018_6384_MOESM3_ESM.pdf]

Description of Additional Supplementary Files

File Name: Supplementary Dataset 1
Description: Data for all figures.
